# The diffuse reduction in spleen density: an indicator of severe acute pancreatitis?

**DOI:** 10.1042/BSR20160418

**Published:** 2017-02-23

**Authors:** Guangdong Shao, Yanmei Zhou, Zengfu Song, Maitao Jiang, Xiaoqian Wang, Xiangren Jin, Bei Sun, Xuewei Bai

**Affiliations:** 1 Department of Pancreatic and Biliary Surgery, First Affiliated Hospital of Harbin Medical University, 23 Youzheng Street, Nangang District, Harbin 150001, Heilongjiang Province, China; 2 Department of Anesthesiology, First Affiliated Hospital of Harbin Medical University, 23 Youzheng Street, Nangang District, Harbin 150001, Heilongjiang Province, China; 3 Department of Hepatobiliary and Pancreatic Surgery, Third Affiliated Hospital of Harbin Medical University, 150 Haping Street, Nangang District, Harbin 150001, Heilongjiang Province, China; 4 Department of Abdominal Ultrasonography, First Affiliated Hospital of Harbin Medical University, 23 Youzheng Street, Nangang District, Harbin 150001, Heilongjiang Province, China

**Keywords:** acute pancreatitis, spleen density, tuftsin, B cell, pyroptosis

## Abstract

We observed that acute pancreatitis (AP) was associated with diffuse reduction in spleen density (DROSD) in some patients. Furthermore, the condition of these patients was more serious, and the potential relationship between DROSD and structural and functional injury of the spleen remained unclear. Therefore, we performed a preliminary exploration of these factors. We analysed pertinent clinical data for AP patients with normal spleen density (control group) and for those with DROSD (reduction group) at the First Affiliated Hospital of Harbin Medical University (June 2013–June 2015). We measured the immunoglobulin M (IgM) B-cells of the AP patients and examined pancreatic and splenic tissues from AP rats with optical microscopy and TEM. The reduction group had a higher acute physiology and chronic health evaluation II (APACHE II) score, a longer length of stay (LOS) and lower serum calcium than the control group. The levels of triglycerides (TG) and total cholesterol (TC) did not differ significantly between the two groups. The percentage of IgM memory B-cells was significantly lower in the DROSD group than in the control group. TEM revealed that the spleen T-lymphocytes were normal in AP rats, but pyroptotic and necrotic spleen B-cells were observed in the severe AP rats. In AP, DROSD was an independent indicator of more severe conditions. Furthermore, spleen B-lymphocytes showed obvious damage at the cellular level, and the immunological function of the spleen was down-regulated when AP was associated with DROSD.

## Introduction

Acute pancreatitis (AP) is a complex abdominal disease that involves an intense local and systemic inflammatory response syndrome (SIRS). Furthermore, severe acute pancreatitis (SAP) is more serious because it is prone to cause complicated multiple organ dysfunction syndrome (MODS), which is one of the main causes of death in patients with SAP [1–3]. Thus, the injury mechanisms and functional protection of the organs far from the pancreas, such as the liver, kidneys and lungs, have been intensively studied by numerous scholars [4–6]. However, although the spleen is an adjacent organ of the pancreas, and its anatomical location is very close to the pancreas, research on spleen injury in the course of AP is still weak. As one of the largest AP centres in Northeast China, more than 200 AP patients are treated in our centre each year. In our long-term clinical practice, we have observed that some AP patients can show a diffuse reduction in spleen density (DROSD) on computed tomography (CT). We initially believed that this finding was only an occasional phenomenon. However, the number of such patients has increased as we have seen more AP patients, and we have observed that the appearance of this phenomenon seems to be related to the condition of AP. Herein, we consulted much of the relevant literature, but only related case reports were found. For example, Arenal et al. [7] reported two cases of AP complicated by splenic infarction; Ray et al. [8] reported a case of SAP in which the patient's spleen was removed due to diffuse splenic infarction; Hernani et al. [9] reported one case of AP complicated by atraumatic splenic rupture; Jiang et al. [10] reported two cases of AP associated with reduction in spleen density, and they proposed that the phenomenon might be related to lipid metabolism. Unfortunately, due to the limitation of sample size, these reports only referred to the phenomenon of splenic injury in AP, but failed to investigate the relevance between the condition of AP and the injury of spleen. Herein, if we can confirm that the occurrence of the DROSD is linked to the condition of AP, our study will provide some clues for the research on the correlation between the condition of AP and the injury of spleen.

In addition, AP can lead to complex immune disturbances during the disease course, characterized by pro-inflammatory and anti-inflammatory responses, which are among of the reasons for the progression of AP [11,12]. When AP occurs, the enhancement of the pro-inflammatory response can stimulate the ability of the body’s immunity to resist infection and to promote repair [13]. However, if the pro-inflammatory response is overactivated, the pro-inflammatory cytokines are uncontrolled and excessively released, resulting in the occurrence and continuous existence of the SIRS, inducing immune disturbance characterized by pro-inflammatory response [11]. In fact, along with the overactivated pro-inflammatory response, anti-inflammatory responses are also activated to resist the excessively activated pro-inflammatory response. For instance, the expression of human leucocyte antigen-DR (HLA-DR) on monocytes is decreased; the synthesis and release of interleukins (ILs)-10, 11 and 1ra, tumour necrosis factor receptor-α (TNFR-α) etc., which have direct anti-inflammatory effects, are increased [14,15]. With progression, compensatory anti-inflammatory response syndrome (CARS) can occur, in which the anti-inflammatory mediators become dominant, which results in immune disturbance characterized by anti-inflammatory response and immune suppression, which renders the body susceptible to infection [12,16,17]. Therefore, maintaining immune homoeostasis is vital for the outcomes of AP. The spleen, compared with the liver, kidneys, lungs etc., is the largest peripheral immune organ and is crucial in regulating immune homoeostasis via its ability to link innate and adaptive immunity [18]. Indeed, patients who underwent splenectomy are significantly susceptible to bacterial infection and, even worse, overwhelming post-splenectomy infection (OPSI), which has been seen worldwide [19,20]. Additionally, Yasuda et al. [21] showed that splenocytes were recruited into the systemic circulation in response to peripheral lymphocyte reduction in experimental SAP. Hughes et al. [22] reported that SAP in a rat model was associated with significant up-regulation of tumour necrosis factor-α (TNF-α) mRNA in splenic mononuclear cells. Norman et al. [23] reported that IL-1β and TNF-α were produced in the spleen in necrotizing pancreatitis induced in young female mice. All of these data demonstrated that the spleen participates in AP regulation of immune homoeostasis. However, studies of the spleen’s immune function in AP remain insufficient, especially when the AP is associated with DROSD. Therefore, if we can figure out its changes, especially the ones of spleen immune function when the spleen density is reduced, the immune regulation theory in AP will be enriched.

Based on the above considerations, we conducted an international, single-centre study enrolling the most AP patients to date with diagnoses of DROSD. The aim of the present study was to determine whether DROSD was related to the severity of AP and whether it was related to structural and functional injury of the spleen.

## Materials and methods

### Patients and experimental design

Based on the classification of AP established in 2012 in the revision of the Atlanta Classification and Definitions by International Consensus (Atlanta International Consensus), we selected patients from the First Affiliated Hospital of Harbin Medical University (June 2013–June 2015) with diagnoses of AP [24]. We excluded patients with past histories of splenic diseases or incomplete medical records. A total of 377 patients met the above criteria and were included in the present study. Within 48 h of hospitalization, we collected the highest acute physiology and chronic health evaluation II (APACHE II) score, total cholesterol (TC), triglycerides (TG) and the lowest concentration of serum calcium (Ca^2+^) of the 377 AP patients. We also recorded their length of stay (LOS).

**Figure 1:**
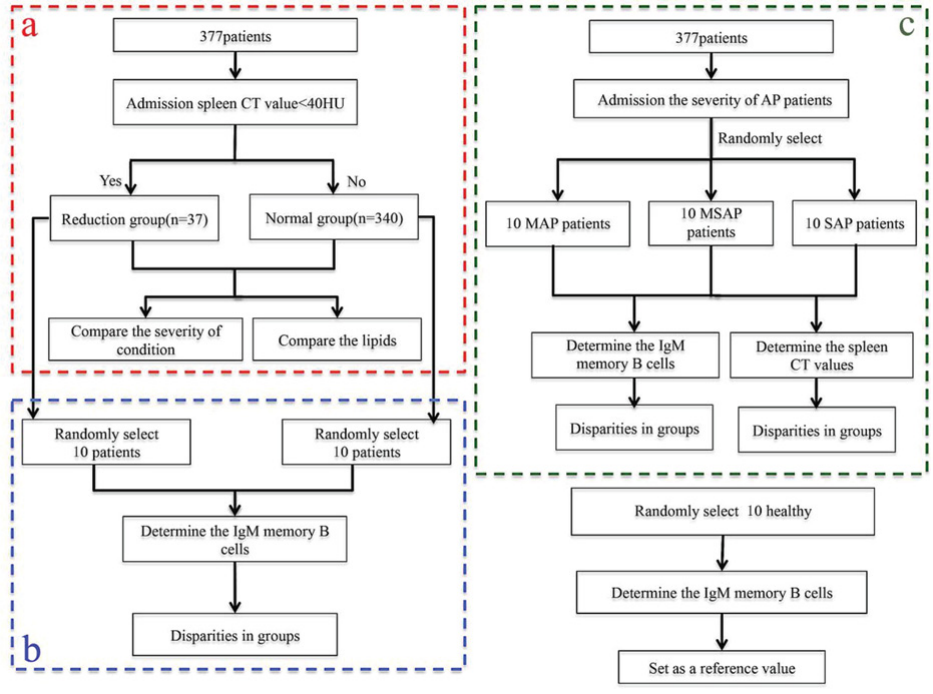
Block flow chart of experimental grouping

**Experiment 1**: According to the spleen CT value, we classified the 377 AP patients into two groups: the control group (40≤ CT value ≤55 HU) and the reduction group (CT value < 40 HU) [25]. To assess the conditions of the patients in the two groups, we analysed the APACHE II score, Ca^2+^ and LOS. We also analysed the correlations of DROSD with TC and TG using logistic regression (Figure 1a).

** Experiment 2**: We randomly selected ten AP patients each from the control group and the reduction group. Then, we drew 10 ml of peripheral venous blood from these selected patients into heparinized tubes for the analysis of immunoglobulin M (IgM) memory B-cells (Figure 1b).

**Experiment 3**: According to the severity of the AP patients’ conditions, we randomly selected patients with diagnoses of mild acute pancreatitis (MAP) (*n*=10), moderately severe acute pancreatitis (MSAP) (*n*=10) and SAP (*n*=10) from the 377 patients. We drew 10 ml of peripheral venous blood from these selected patients and from ten randomly selected healthy volunteers into heparinized tubes for the analysis of IgM memory B-cells. We evaluated the immunological function of the spleen based on the percentage of IgM memory B-cells. In addition, we determined the AP patients’ spleen CT values and compared the differences among these three groups (Figure 1c).

The present study was approved by the institutional review board of the First Affiliated Hospital of Harbin Medical University (study protocol No. 201511), and all samples were collected with the informed consent of the patients. The patient blood samples were analysed by the Department of Medical Laboratory at the First Affiliated Hospital of Harbin Medical University, and abdominal CT scans of the patients were performed by the Department of Radiology at the First Affiliated Hospital of Harbin Medical University.

### Measurement of the spleen CT values for AP patients

The spleen CT values of the AP patients were determined by the Department of Radiology of the First Affiliated Hospital of Harbin Medical University under the same scanning parameters. Briefly, the location conditions included 400 mm of the scanning range, 180 degrees of the rotation angle, 120 kV of the tube voltage and 50 mA of the tube current. The axial scanning conditions included 300 mm of the scanning length, 3 mm of the layer thickness, 120 kV of the tube voltage and 80 mA of the tube current. The scanning dose conditions included 21.1 mGy of the computed tomography dose index (CTDI), 11.3 s of the time and 720.0 mGy·cm of the dose–length product (DLP).

### Flow cytometric analysis of IgM memory B-cells

Peripheral blood mononuclear cells (PBMCs) were isolated from heparinized peripheral venous blood by Lymphoprep gradient centrifugation (TBD Sciences, Tianjin, China), and they were incubated with fluorescent label-conjugated monoclonal antibodies at saturating concentrations (anti-human CD22, anti-human CD27 and anti-human IgM: 20 μl/100 μl experimental sample of 1 × 10^6^ cells; anti-human IgD: 5 μl/100 μl experimental sample/1 × 10^6^ cells) for analysis with a FACS Aria Flow Cytometer (BD Biosciences, U.S.A.), as previously described [26]. FITC-conjugated anti-human CD22, PE-conjugated anti-human CD27, APC-conjugated anti-human IgM and PerCP-Cy™5.5-conjugated anti-human IgD were obtained from BD Biosciences. Analysis was performed on 1000 memory B-cells (CD22^+^CD27^+^) per sample. Within CD22^+^CD27^+^ B-cells, IgM memory B-cells were defined as IgM-only, IgM^+^ IgD^+^ and IgD-only [27].

### Animals and experimental design

The University Animal Ethics Committee approved all animal care and experimental protocols (study protocol No. 2015006). Seventy-five male Wistar rats weighting 250–300 g were purchased from the Animal Research Center at the Second Affiliated Hospital of Harbin Medical University in Harbin, China. The rats were fed rodent chow, and water was provided under standard conditions (12-h light/dark cycles). The rats were randomly assigned to three groups: sham, MAP and SAP. Each group consisted of 25 rats. The rats were fasted overnight prior to surgery and were anesthetized via intraperitoneal injection of 40 mg/kg pentobarbital. The sham operation included only laparotomy and puncture of the duodenal wall. MAP and SAP were induced using the methods described previously by Cuthbertson et al. [28] and Bai et al. [29]. Briefly, we performed a midline laparotomy and introduced a blunt 27-gauge needle into the distal end of the biliopancreatic duct via puncture of the duodenal wall. Prior to injection, the proximal bile duct was temporarily occluded at the porta hepatis by a vascular clamp, and the distal duct was blocked by the placement of a titanium aneurysm clip. Sodium taurocholate (MAP: 1.5%; SAP: 3.5%; 20 mg/kg, Sigma–Aldrich, Shanghai, China) was infused at a pressure of 5 kPa controlled using a sphygmomanometer. The needle, vascular clamp and titanium clip were removed after 5 min of the injection. The duodenal and abdominal wounds were closed, and the rats recovered on heated pads.

Rats in each group were chosen randomly to be killed at 6, 12, 24, 48 or 72 h after surgery. The peripheral venous blood was centrifuged at 3000 rpm for 10 min, and the supernatant serum was collected and stored at −80°C [30]. In addition, the mass of the spleen was measured using a micro-electronic balance (FA-1004, Shanghai Precision Instrument Co., Ltd., China), and the volume of the spleen was measured using a precision cylinder. The density of the spleen was calculated using the formula (*ρ*=*m*/*v*). The pancreatic and splenic tissues were then fixed in formalin or glutaraldehyde.

### Evaluation of the splenic haemoperfusion of the rats

Fifteen male Wistar rats weighting 250–300 g were randomly assigned to three groups: sham (*n*=5), MAP (*n*=5) and SAP (*n*=5). MAP and SAP were induced using the methods described above. One rat in each group was chosen randomly to evaluate the splenic haemoperfusion with ultrasound at 6, 12, 24, 48 or 72 h after surgery. Briefly, we initially identified the spleens of the rats with grey-scale ultrasound, and on the basis of these findings, we firstly evaluated the splenic haemoperfusion with colour Doppler ultrasound. Then, we switched the sonographic unit to the contrast-tuned imaging mode, and an ultrasound contrast agent (SonoVue, Bracco International, Amsterdam, the Netherlands) at a dose of 0.1 ml/kg was administered as a bolus into the caudal vein of the rat, followed by a 0.15 ml saline flush [31,32]. Immediately after injection, continuous scanning of the spleen was conducted to evaluate further the splenic haemoperfusion.

### Measurement of the tuftsin concentration in the serum of AP rats

The tuftsin concentration in the serum of rats was determined by HPLC, as described previously by Amoscato et al. [33]. Detection and quantification of analytes were performed on a LC apparatus (LC-20AT) under suitable chromatographic conditions, indicating that the standards and samples were separated on a YMC-Triart C_18_ (Shimadzu, 250×4.6 mml.D S-5 μm, 12 nm) column by isocratic elution using a mobile phase consisting of methanol (≥99.9%, J&K Scientific, Beijing, China) and heptafluorobutyric acid (C_4_HF_7_O_2_) solution (0.0023 mol/l, Sinopharm Chemical Reagent Co., Ltd, Shanghai, China) (35:65, v/v) at a constant flow rate of 1.0 ml/min. All of the analyses were monitored at a wavelength of 214 nm at an ambient temperature. The chromatogram run time was 30 min.

To obtain the calibration graph and the regression, a stock solution (0.4 mg/ml) of tuftsin acetate salt hydrate was prepared by dissolving 4 mg of compound in 10 ml of methanol. Working solutions (40, 8, 4, 2, 1, 0.4 and 0.2 μg/ml) were prepared by appropriate serial dilution of the stock solution with methanol. These working solutions were analysed to obtain their peak areas, and then the calibration graph was drawn to obtain the regression equation with different concentrations of the working solutions and their corresponding peak areas.

To obtain the peak area of the serum tuftsin in rats, 1 ml of serum was diluted with 3 ml of (NH_4_)_2_CO_3_ (0.1 mol/l, Amresco, U.S.A.) and digested with 1 mg of type Ι trypsin [30]. The digestion was incubated at 37°C for 1 h, and 17.5 ml of 95% alcohol was added. The mixture was then incubated at 80°C for 15 min. After cooling on ice, the mixture was centrifuged at 3000 rpm for 30 min at 4°C. The supernatant containing tuftsin was decanted into a 25 ml distilling flask, and the solvent was evaporated using a rotary evaporator. The dry residue was gathered and dissolved in a mixture of acetonitrile and water (200 μl, 1:1, v/v). Then, the supernatant was analysed by HPLC to obtain the peak area.

### Histological examination

Formalin-fixed tissues of the pancreas and spleen were embedded in paraffin, sectioned, stained with haematoxylin and eosin (HE) and examined by light microscopy. The pathological scoring of the pancreatic tissue was performed by two professional histologists according to the criteria described by Schmidt et al. [34]. We scored the pancreatic tissues for oedema, acinar necrosis, haemorrhage and fat necrosis, inflammation and perivascular infiltration [35].

### TEM analysis

TEM analysis was performed to observe the changes in the spleen. Splenic tissues were double fixed in 2.5% glutaraldehyde and 1% osmic acid for 2 h and then rapidly stained with 2% acetic acid U, dehydrated in a graded series of alcohol and acetone, embedded in Epon 812 and cut with a Reichert-Jung ultramicrotome. The specimens were double stained with acetic acid U and lead citrate solution and examined with a HITACHIH-7650 operating at 80 kV [36].

### Statistical analysis

The results are presented as the mean ± S.D. The data were analysed by one-way ANOVA followed by a Student–Newman–Keuls test, with Bonferroni correction for multiple comparisons. *t*-Statistics were used for data with a continuous and normal distribution, and the rank-sum test was used for abnormally distributed data. Categorical variables were compared using chi-square tests. Logistic regression analysis was performed to identify influencing factors. All analyses used a two-sided test using SPSS 19.0 software. The level of significance was set for *P*-values less than 0.05.

## Results

There were no significant differences in age or sex among the groups (Table 1 and Table 2).

**Table 1:** Age difference analysis: the age of the AP patients in experiment 1 and the age of the AP patients in experiment 2

	Control group		Reduction group		Mann–Whitney U test
	(*n=340*)		(*n=37*)		(*P-value*)
Age (years)	44.31 ± 12.88		45.65 ± 10.56		0.517
**A: Grouped by AP patients’ spleen density**					
	Control group		Reduction group	Volunteers	Mann–Whitney U test
	(*n=10*)		(*n=10*)	(*n=10*)	(*P-value*)
Age (years)	52.60 ± 11.82		47.00 ± 11.96	47.30 ± 6.26	0.382
**B: Grouped by AP patients' condition**					
	MAP group	MSAP group	SAP group	Volunteers	Mann–Whitney U test
	(*n=10*)	(*n=10*)	(*n=10*)	(*n=10*)	(*P-value*)
Age (years)	45.20 ± 13.27	45.60 ± 15.02	41.50 ± 10.68	47.30 ± 6.26	0.835

**Note:** Statistical analysis was performed with the Mann–Whitney U test. Control group: AP patients with normal spleen density; Reduction group: AP patients with DROSD; Volunteers: healthy volunteers; MAP group: patients with a diagnosis of MAP; MSAP group: patients with a diagnosis of MSAP; SAP group: patients with a diagnosis of SAP. The level of significance was set for *P*-values less than 0.05.

**Table 2:** Sex difference analysis: the sex of the AP patients in experiment 1 and the sex of the AP patients in experiment 2

Sex	Control		Reduction		Chi-square test
	(*n=340*)		(*n=37*)		(*P*-value)
M	235 (69.1%)		21 (56.8%)		0.126
F	105 (30.9%)		16 (43.2%)		
**A: Grouped by AP patients’ spleen density**					
	Control group		Reduction group	Volunteers	Chi-square test
	(*n=10*)		(*n=10*)	(*n=10*)	(*P* -value)
M	7		6	7	0.861
F	3		4	3	
**B: Grouped by AP patients’ condition**					
	MAP group	MSAP group	SAP group	Volunteers	Chi-square test
	(*n=10*)	(*n=10*)	(*n=10*)	(*n=10*)	(*P*-value)
M	6	4	5	7	0.568
F	4	6	5	3	

**Note:** Statistical analysis was performed with the Chi-square test. Control group: AP patients with normal spleen density; Reduction group: AP patients with DROSD; Volunteers: healthy volunteers; MAP group: patients with a diagnosis of MAP; MSAP group: patients with a diagnosis of MSAP; SAP group: patients with a diagnosis of SAP; M: male; F: female. There was no significant difference in sex. The level of significance was set for *P* -values less than 0.05.

### SAP patients had lower spleen CT values than MAP and MSAP patients

**Figure 2:**
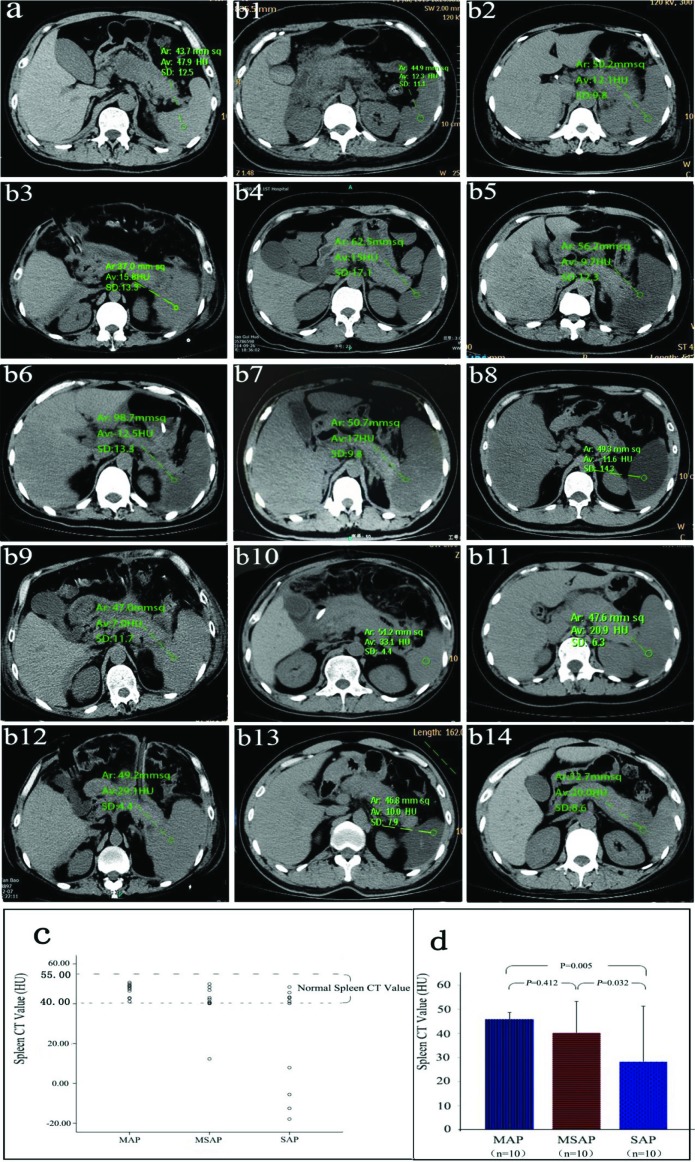
Abdominal CT scans of AP patients **(a)** Abdominal CT scan of an AP patient with normal spleen density. The CT value of the spleen was 47.9 HU. **(b1-b14)** Abdominal CT scans of AP patients with DROSD. The CT values of the spleen (12.3, 12.1, 15.8, 15, −9.7, −12.5, 17, −11.6, 7.0, 33.1, 20.9, 29.1, 10.0 and 20.0 HU respectively) were lower than the normal values (40–55 HU). **(c)** Individual spleen CT values in the MAP group (*n*=10), MSAP group (*n*=10) and SAP group (*n*=10). The CT values between the two dotted lines are the normal range of spleen CT values. **(d)** The average spleen CT value of the SAP group was significantly less than those of the MAP and MSAP groups. The level of significance was set for *P*-values less than 0.05.

Clinically, we observed that some AP patients were complicated by DROSD, and we collected 37 cases of such patients in the present study. As shown in representative images in Figure 2a and b, compared with the normal spleens of AP patient (Figure 2a), the spleens with reduced density (Figure 2b) presented a greyer image, and their CT values were less than the normal values (12.3, 12.1, 15.8, 15.0, −9.7, −12.5, 17.0, −11.6, 7.0, 33.1, 20.9, 29.1, 10.0 and 20.0 HU compared with 47.9 HU). At the same time, the spleen CT values of the MAP, MSAP and SAP patients in experiment 3 were collected and compared. As shown in Figure 2c, all of the spleen CT values of the patients in the MAP group were in the normal range. However, one patient**’**s spleen CT value in the MSAP group and four patients’ spleen CT values in the SAP group were less than the normal value. As shown in Figure 2d, the average spleen CT value of the SAP group was significantly less than that of the MAP and MSAP groups (23.36 ± 27.02 compared with 46.69 ± 3.34 and 40.41 ± 10.51; *P*<0.05). However, the difference in the average spleen CT value between the MAP group and the MSAP group was not significant (46.69 ± 3.34 compared with 40.41 ± 10.51; *P*=0.412).

### AP patients with DROSD had more serious conditions than those with normal spleen density

APACHE II, serum calcium values and LOS were collected to assess the conditions of AP patients [37]. As shown in Table 3, the patients in the reduction group had significantly higher APACHE II scores and longer LOS than those in the control group (*P*<0.001). Furthermore, the concentration of serum calcium was significantly less in the reduction group than in the control group (*P*=0.001). Thus, the conditions of the AP patients with DROSD were more serious than those of the patients with normal spleen density.

**Table 3:** Assessment of AP patients’ condition

	Control group	Reduction group	Mann–Whitney U test
	(*n*=340)	(*n*=37)	(*P*-value)
APACHE II	5.87 ± 4.18	9.22 ± 6.05	0.000
Serum calcium (mmol/l)	2.09 ± 0.21	1.99 ± 0.16	0.001
LOS (d)	15.13 ± 15.11	23.95 ± 18.49	0.000

**Note:** The data are expressed as the mean ± S.E.M., and statistical analysis was performed with the Mann–Whitney U test. Control group: AP patients with normal spleen density; Reduction group: AP patients with DROSD. The level of significance was set for *P* -values less than 0.05.

### SAP rats had lower spleen density than MAP rats

**Figure 3:**
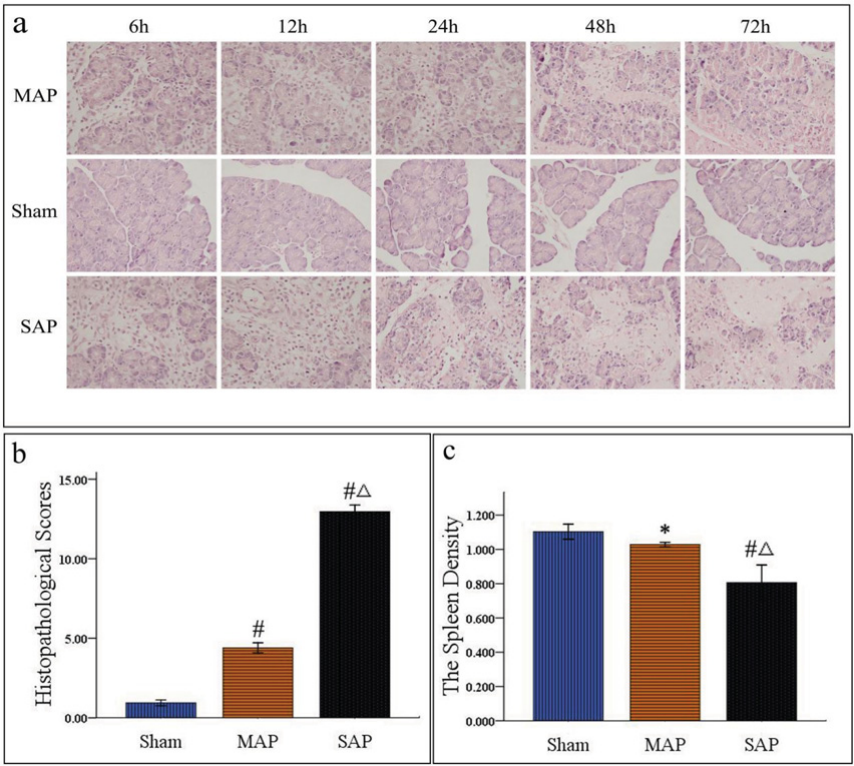
(a) Representative morphological features of the rat pancreas (×40 magnification). Compared with the normal morphology of the sham group, the MAP group exhibited mild oedema and a small amount of inflammatory cell infiltration at 6, 12, 24, 48 and 72 h. By contrast, the SAP group exhibited obviously serious oedema, extensive necrosis, focal haemorrhage, and inflammatory cell infiltration at 6, 12, 24, 48 and 72 h. **(b)** Pathological scoring of pancreatic tissues (*n*=25 respectively). The average pathological score was significantly higher in the SAP group than the sham group (*P*<0.001) or MAP group (*P*<0.001). **(c)** Spleen density of the rats (*n*=25 respectively). The average spleen density was significantly lower in the SAP group than in the sham group (*P*<0.001) or MAP group (*P*<0.001). The differences in spleen density between the MAP group and sham group were not significant (*P*>0.05). ^#^*P*<0.001 compared with the sham group, ^Δ^*P*<0.001 compared with the MAP group, *P>0.05 compared with the sham group.

To confirm further the correlation between spleen density and the severity of AP, the spleen density of the rats was measured, and the pancreatic tissues of the rats were scored for oedema, acinar necrosis, haemorrhage, fat necrosis, inflammation and perivascular infiltration [35]. As shown in representative images in Figure 3a, compared with the normal morphology in the sham group, the MAP group exhibited mild oedema and a small amount of inflammatory cell infiltration at 6, 12, 24, 48 and 72 h. However, the SAP group exhibited serious oedema, extensive necrosis, focal haemorrhage and inflammatory cell infiltration. As shown in Figure 3b, the average pathological score of the SAP group was significantly higher than those of the sham group and MAP group (12.96 ± 1.02 compared with 0.94 ± 0.44 and 4.40 ± 0.76; *P*<0.001). In contrast, the average spleen density was significantly lower in the SAP group than in the sham and MAP groups (0.81 ± 0.25 compared with 1.10 ± 0.11 and 1.03 ± 0.03; *P*<0.001; Figure 3c). However, the difference in spleen density between the MAP group and the sham group was not significant (1.03 ± 0.03 compared with 1.10 ± 0.11; *P*>0.05). From these data, we found that the conditions of the rats in SAP group were more serious, but the spleen density of the rats in the SAP group was less.

### There was larger spleen volume in the SAP group than in the sham and MAP groups

**Figure 4:**
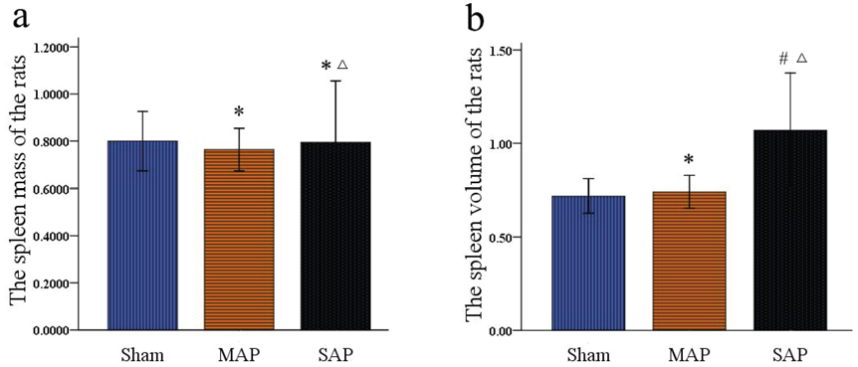
The average mass and volume of the rats spleen (*n*=25 respectively) **(a)** There were no differences in the average mass of the spleen among the three groups. *P>0.05 compared with the sham group, ^Δ^*P*>0.05 compared with the MAP group. **(b)** The average volume of the spleens in the SAP group was significantly greater than those in the sham group and the MAP group. However, the difference between the MAP group and the sham group was not significant. *P>0.05 compared with the sham group, ^#^*P*<0.05 compared with the sham group, ^Δ^*P*<0.05 compared with the MAP group.

To explore the reasons for the spleen density changes in rats, the mass and volume of the spleen were analysed. As shown in Figure 4a, there were no differences in the average mass of the spleen among the three groups (sham compared with MAP: 0.80 ± 0.31 compared with 0.76 ± 0.22, *P*=0.765; sham compared with SAP: 0.80 ± 0.31 compared with 0.79 ± 0.63, *P*=0.967; MAP compared with SAP: 0.76 ± 0.22 compared with 0.79 ± 0.63, *P* =0.796). In contrast, the average volume (Figure 4b) of the spleens in the SAP group (1.07 ± 0.74) was significantly greater than those in the sham group (0.72 ± 0.22; *P*=0.009) and the MAP group (0.74 ± 0.21; *P*=0.015); however, the difference between the MAP group and the sham group was not significant (*P*=0.865). Thus, the changes in the spleen density of the rats were related to the spleen volume instead of the spleen mass.

### Splenic haemoperfusion decreased in SAP rats

**Figure 5:**
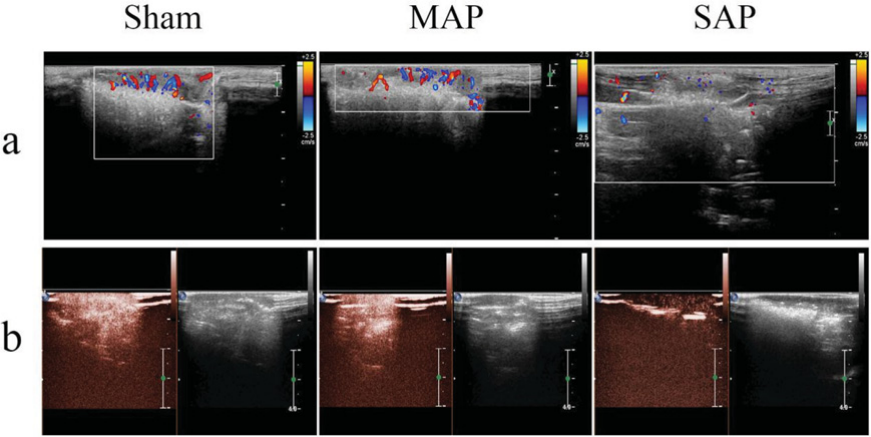
The splenic haemoperfusion of the rats **(a)** Representative sonogram of the splenic haemoperfusion on colour Doppler ultrasound. The blood flow signals of spleens did not differ between the sham group and MAP group of AP rats, and all of them presented with clustered and locally dense blood flow signals; however, the splenic haemoperfusion decreased in SAP rats, and their blood flow signals of spleens showed a punctate or linear pattern. (**b)** Representative sonogram of the splenic haemoperfusion on ultrasound contrast imaging. There was no significant differences in the visible signal intensity of the spleen between the sham and MAP groups; however, the splenic haemoperfusion decreased in SAP rats, and their visible signal intensity of the spleen was lower.

As shown in the representative sonogram of colour Doppler ultrasound (Figure 5a), the blood flow signals of spleens did not differ between the sham group and MAP group of AP rats, and all of them presented with clustered and locally dense blood flow signals; however, compared with the sham group, the blood flow signals of spleens in the SAP group showed a punctate or linear pattern at 24, 48 and 72 h after surgery, but no obvious differences were found at 6 and 12 h. Furthermore, on representative ultrasound contrast imaging (Figure 5b), no significant differences in the visible signal intensity of the spleen were observed between the sham and MAP groups; however, compared with the sham group, the visible signal intensity of the spleen was lower at 24, 48 and 72 h after surgery, but no obvious differences were found at 6 and 12 h. From these data, we could see that the splenic haemoperfusion in SAP rats decreased.

### No correlation between DROSD and blood lipids in AP patients

**Figure 6:**
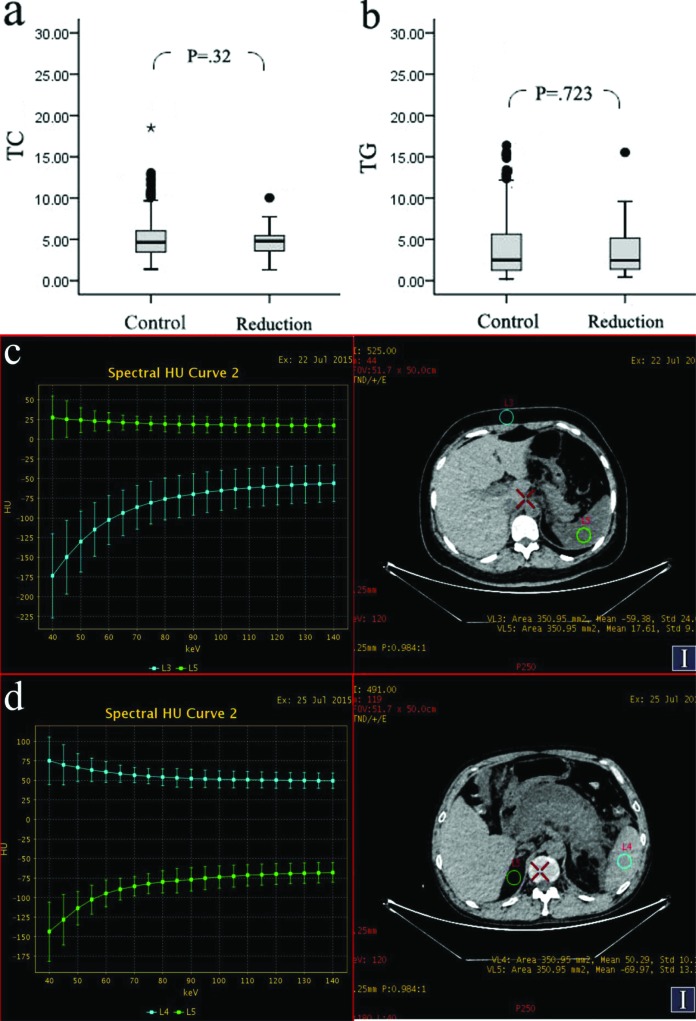
Correlation between diffuse reduction of spleen density and blood lipid levels **(a and b)** TC and TG in the reduction (*n*=37) and control (*n*=340) groups were analysed by logistic regression. **(a)** No difference in TC was observed between the reduction group and the control group. (**b)** No difference in TG was observed between the reduction group and the control group. **(c, d)** Spectral CT images of AP patients. **(c)** The spectral CT curves of the spleen (the green line) and abdominal fat (the sky blue line) exhibited opposing trends for patients with AP with DRSOD. **(d)** The spectral CT curves of the spleen (the sky blue line) and abdominal fat (the green line) exhibited opposing trends for patients with AP with normal spleen density, as observed for AP patients with DROSD.

Because DROSD in AP might be related to high blood lipid levels, we analysed the correlations of TC and TG with spleen density by logistic regression [10]. As shown in Figure 6a and b, TC and TG did not differ between the control and reduction groups of patients with AP (4.69 ± 1.95 compared with 5.26 ± 2.88, *β* = −0.096, *P*=0.32, *P*>0.05; 3.82 ± 3.15 comapred with 4.12 ± 4.22, *β* = −0.019, *P*=0.723, *P*>0.05). In addition, Gemstone spectral imaging (GSI) analysis of AP patients revealed that the spectral CT curves of the spleen and abdominal fat exhibited opposite trends, with no difference between the control group and the reduction group (Figure 6c and d). These results suggested that DROSD in AP was not caused by the accumulation of lipids in the spleen.

### The different pathological changes of spleens in the MAP and SAP groups

**Figure 7:**
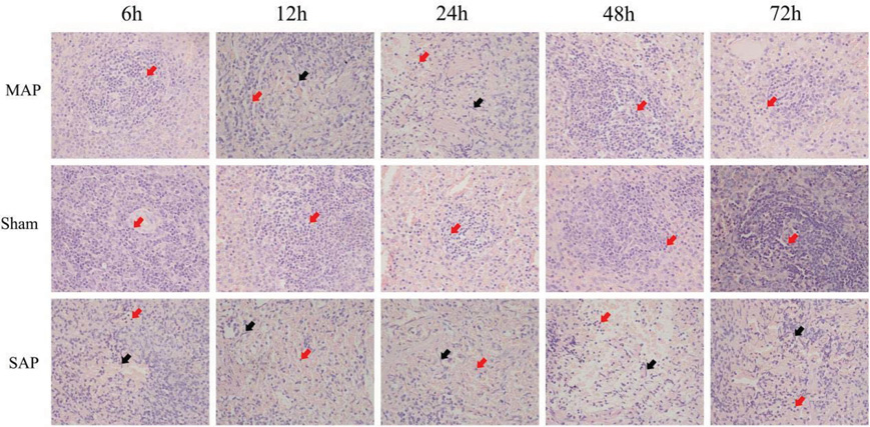
Representative morphological features of the rat spleen (×40 magnification) Compared with the normal morphology observed in the sham groups, the MAP group exhibited a small amount of fibroblast infiltration at 12 and 24 h, but the differences were not significant at 6, 48 and 72 h. In addition, compared with the sham group, the SAP groups exhibited a small amount of fibroblast (black arrows) infiltration at 6 h and obvious fibroblasts infiltration and a decrease in lymphocytes (red arrows) at 12, 24, 48 and 72 h.

To analyse the pathological impairment of the spleen in AP, the splenic tissues of the rats were examined by optical microscopy. Representative pathological images of splenic tissue obtained by optical microscopy are presented in Figure 7. Compared with the normal morphology of the sham group, the MAP group exhibited a small amount of fibroblast infiltration at 12 and 24 h, but the differences between the groups were not significant at 6, 48 and 72 h. However, the SAP group exhibited a small amount of fibroblast infiltration at 6 h and obvious fibroblast infiltration and a decrease in lymphocytes at 12, 24, 48 and 72 h.

### More obvious damage to rat splenic B-lymphocytes

**Figure 8:**
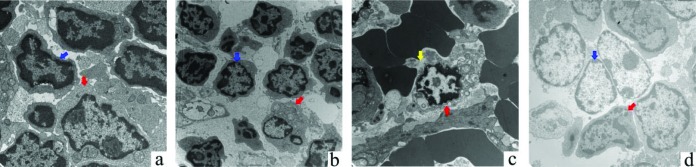
Representative ultrastructural features of the rat spleen **(a)** Normal T-lymphocyte (blue arrow) and B-lymphocyte (red arrow). (**b)** Normal T-lymphocyte (blue arrow) and the apoptotic B-lymphocyte (red arrow). (**c)** Pyroptotic B-lymphocyte (red arrow). The yellow arrow indicates the site of membrane rupture. **(d)** Normal T-lymphocyte (blue arrow) and necrotic B-lymphocyte (red arrow).

To further observe the ultrastructural changes of the lymphocytes, rat spleens were examined by TEM. Thin sections of splenic tissue samples from the sham group revealed normal B-lymphocytes and T-lymphocytes characterized by nuclear regularity and a complete cell membrane (Figure 8a). In the MAP group, apoptotic B-lymphocytes were observed, and these B-lymphocytes were characterized by nuclear condensation, chromatin condensation and swollen mitochondria; however, compared with the sham group, there was no difference in T-lymphocytes, which were characterized by a clear and complete cell structure and regular nuclear morphology (Figure 8b). In the SAP group, we observed pyroptotic B-lymphocytes characterized by nuclear condensation and a ruptured cell membrane (Figure 8c). We also observed necrotic spleen B-lymphocytes, which were characterized by lytic organelles, a disrupted membrane and a naked nucleus, but there was no difference in T-lymphocytes compared with the sham group (Figure 8d). These results suggest that the spleen B-lymphocytes are obviously damaged in AP.

### The immunological function of the spleen in AP patients with DROSD down-regulated

**Figure 9:**
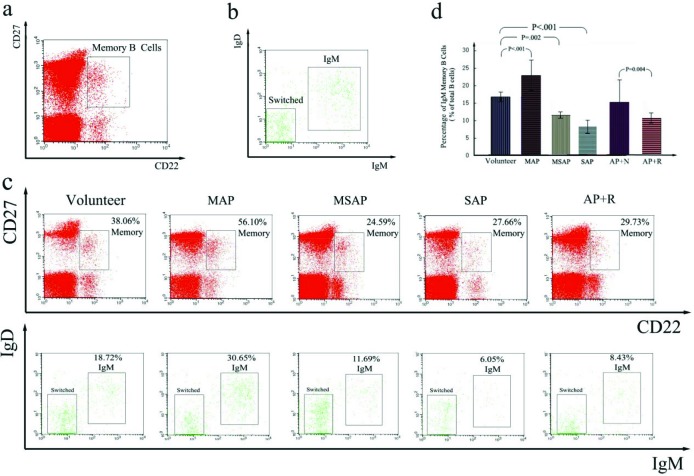
Evaluation of splenic immune function by B lymphocyte. PBMCs were stained with Abs to CD22, CD27, IgM and IgD and analysed by four-colour flow cytometry. The data are presented as density plots. Phenotypic analysis of B-cells in human peripheral venous blood (**a)** identification of CD27^+^ memory B-cells from CD22^+^ B-cells; (**b)** two populations of memory B-cells were identified: IgM memory B-cells (IgM-only, IgD-only and IgM^+^ IgD^+^) and switched memory B-cells, which do not express both IgM and IgD. **(c)** PBMCs from the enrolled patients were stained and analysed as described in (**a**) and (**b**). At top, the expression of CD22 and CD27 is shown. The numbers indicate the percentage of memory B-cells, calculated as a percentage of CD22+ B-cells. At bottom, IgM and switched memory B-cells were identified as described in (**a**) and (**b**). The numbers indicate the percentage of IgM memory B-cells, calculated as a percentage of CD22^+^ B-cells. **(d)** The percentage of IgM memory B-cells (% of CD22^+^ B-cells) in the different groups was analysed by flow cytometry (*n*=10 respectively). The percentage of IgM memory B-cells was significantly higher in MAP patients compared with the group of healthy volunteers (*P*<0.001) but was significantly lower in MSAP (*P*=0.002) and SAP (*P*<0.001) patients. Moreover, the patients with DROSD had a low percentage of IgM memory B-cells compared with the patients with AP with normal spleen density. **AP+N:** AP patients with normal spleen density. **AP+R:** AP patients with diffuse reduction of spleen density.

Two populations of B-cells have been identified in human PBMCs: mature and memory B-cells, which can be distinguished by the combined use of CD22 and CD27. CD22 is a B-cell marker, and CD27 is expressed on memory B- and T-cells [26]. Mature B-cells are CD27^−^, and circulating memory B-cells are CD27^+^ (Figure 9a). Based on the relative expressions of IgM and IgD, memory B-cells can be further subdivided into IgM memory (an IgM-only subset, an IgM^+^IgD^+^ subset and a minor IgD-only subset) and switched memory B-cells (an IgM^−^IgD^−^ subset) (Figure 9b).

To evaluate the immunological function of the spleen in AP patients, we measured the percentage of IgM memory B-cells in the peripheral venous blood of healthy volunteers and AP patients (Figure 9c). The percentage of IgM memory B-cells (Figure 9d) was 16.6 ± 1.34% in healthy volunteers, but it was significantly lower in the patients with MSAP and SAP (11.75 ± 0.91%, *P*=0.002; 8.27 ± 1.91%, *P*<0.001). In contrast, the percentage of IgM memory B-cells was significantly higher in patients with MAP (22.97 ± 4.38%, *P*<0.001), suggesting that the immunological function of the spleen exhibited a biphasic alteration in AP. In addition, we also compared the percentages of IgM memory B-cells in ten patients with AP associated with DROSD and ten patients with AP associated with a normal spleen density. As shown in Figure 9d, the patients with DROSD had a lower percentage of IgM memory B-cells than the patients with normal spleen density (10.64 ± 1.47 compared with 15.18 ± 6.29, *P*=0.004). Moreover, the percentage of IgM memory B-cells in AP patients with DROSD was close to the percentage of IgM memory B-cells in MSAP patients and SAP patients. These results suggested the down-regulation of the immunological function of the spleen in AP patients with DROSD.

### The immunological function of the rat spleen exhibited biphasic and phasic alteration

**Figure 10:**
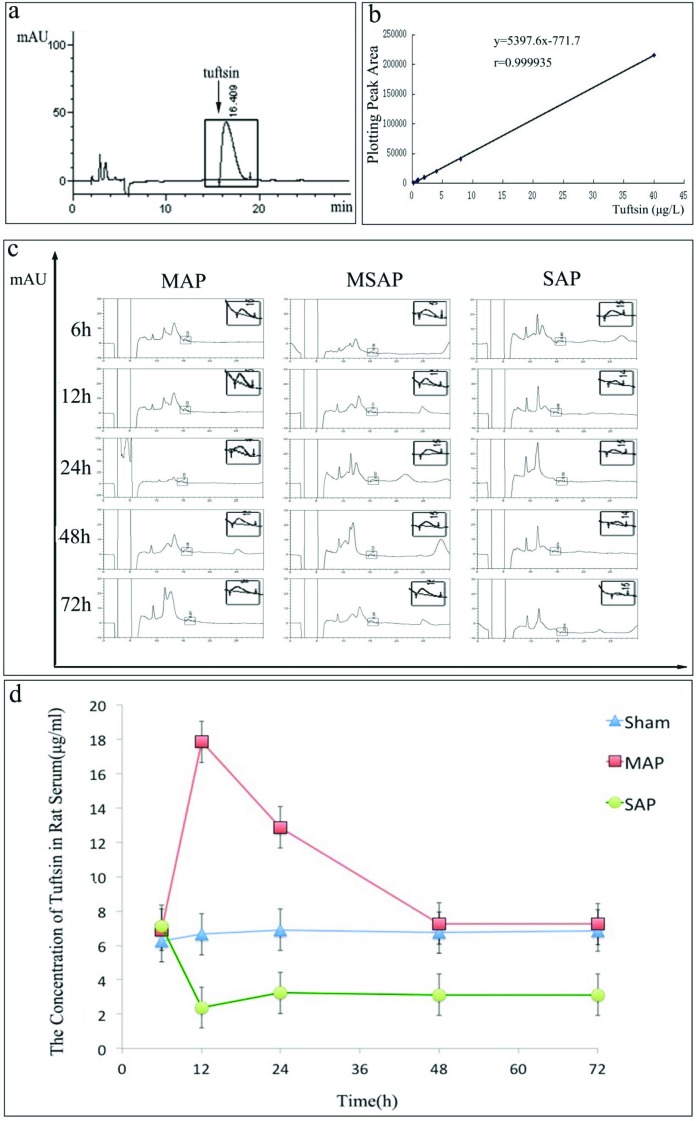
Evaluation of splenic immune function by tuftsin. (**a**) Chromatogram of tuftsin acetate salt hydrate. The peak corresponding to tuftsin was detected at approximately 16 min when the working solution of tuftsin acetate salt hydrate was analysed. **(b)** Tuftsin calibration curve. The relationship between the concentration of tuftsin (*x*) and the plotted peak area of tuftsin (*y*) was linear over the range 0.2–40 μg/ml. The equation for the calibration graph was *y* =5.3976*x–*771.7, and the correlation coefficient was 0.9999. **(c)** Representative chromatogram of tuftsin in rats. Compared with the sham group, 12 and 24 h after the induction of MAP, the area representing the concentration of tuftsin in rat serum increased significantly, whereas no obvious difference was observed at 6, 48 or 72 h. At 6 h after the induction of SAP, no obvious change was observed in the area of tuftsin, but an obvious decrease in the area of tuftsin was observed at 12, 24, 48 and 72 h. **(d)** The trend of the change in the tuftsin concentration in rat serum. Compared with the sham group, the tuftsin concentration in rat serum in the MAP group did not differ at 6 h after model induction but was obviously higher at 12 and 24 h and returned to normal at 48 and 72 h. In addition, compared with the sham group, the tuftsin concentration in the SAP group did not differ at 6 h after model induction but was significantly lower at 12, 24, 48 and 72 h. Recovery of the tuftsin concentration in SAP rats was not observed within the study period.

To evaluate the immunological function of the spleen in AP rats, we detected the tuftsin concentration in the serum [38,39]. As shown in Figure 10a, when the working solution was analysed, the peak area of tuftsin acetate salt hydrate was detected at approximately 16 min, similar to previous studies. Then, a regression equation was obtained from the points corresponding to the seven concentrations of the working solutions (*x*) and their peak areas (*y*) as *y*=5.3976*x*−771.7, with a correlation coefficient of 0.9999. Furthermore, its calibration graph showed that the peak area of tuftsin and the concentration of tuftsin were linear over the range 0.2–40 μg/ml (Figure 10b). Thus, we could obtain the tuftsin concentration in rat serum by the regression equation. As shown in the representative images in Figure 10c, compared with the normal peak area of serum tuftsin in the sham group, the MAP group exhibited a larger size of the peak area at 12 h and 24 h. However, the SAP group exhibited a smaller size at 12, 24, 48 and 72 h. Furthermore, as shown in Figure 10d and Table 4, the tuftsin concentration in serum was significantly higher in the MAP group than that in the sham group at 12 and 24 h (*P*<0.001). However, no differences were observed at 6, 48 and 72 h (6 h: *P*=0.634; 48 h: *P*=0.705; 72 h: *P*=0.786). In contrast with the MAP group, the tuftsin concentration in serum was significantly lower in the SAP group than in the sham group at 12, 24, 48 and 72 h (12 h: *P*=0.003; 24 h: *P*=0.011; 48 h: *P*=0.012; 72 h: *P*=0.010) after surgery. However, there were no obvious differences in the tuftsin concentration between the two groups at 6 h (*P*=0.524). These results suggested that the immunological function of the spleen in AP rats exhibited biphasic and phasic alteration.

**Table 4:** The concentration of tuftsin in rat serum

	6 h (μg/ml)	12 h (μg/ml)	24 h (μg/ml)	48 h (μg/ml)	72 h (μg/ml)
	(*n=5*)	(*n=5*)	(*n=5*)	(*n=5*)	(*n=5*)
Sham	6.239 ± 0.925	6.643 ± 1.007	6.902 ± 0.116	6.741 ± 0.768	6.854 ± 0.480
MAP	6.906 ± 0.376^Δ^	17.840 ± 6.490^#^	12.864 ± 4.148^#^	7.271 ± 1.357^Δ^	7.235 ± 0.949^Δ^
SAP	7.127 ± 0.938^Δ^	2.380 ± 1.135*	3.228 ± 0.863*	3.118 ± 1.761*	3.117 ± 1.471*

**Note:** Data are expressed as the mean ± S.E.M., and statistical analysis was performed with the Student–Newman–Keuls test. *P<0.05 compared with the sham group, ^#^*P*<0.001 compared with the sham group; ^Δ^*P*>0.05 compared with the sham group.

## Discussion

AP can lead to complex immune disturbances during the disease course, and the spleen, which is the largest peripheral immune organ, participates in AP regulation of immune homoeostasis. In the present study, we found that in AP the spleen could be changed, which was characterized by diffuse reduction in density morphologically, and this phenomenon was related to the condition of AP. Moreover, we also demonstrated that the spleen could be impaired ultrastructurally, which was characterized by impaired B-lymphocytes. Thus, the clinical significance , the mechanism and the changes of the immune function of the DROSD in AP are worthwhile to be discussed.

The spleen is an abdominal parenchymal organ of the human body, and a healthy spleen has stable density on CT scans (the CT value is 40–55 HU) [10,26]. However, we recently observed that AP could be associated with DROSD in some patients clinically (Figure 2b). The spleen CT values of these patients were significantly decreased and even negative. Herein, we conducted experiments and compared the spleen CT values of AP patients in MAP, MSAP and SAP groups. We found that the average spleen CT value of the patients in the SAP group was significantly less than that in the MAP and MSAP groups (Figure 2d), suggesting that the decrease in spleen density was related to the severity of AP. Moreover, we observed that these patients had longer lengths of stay (Table 3), and the treatment of these patients was more difficult and challenging work for us. Thus, we analysed the APACHE II score and the concentration of serum calcium in two groups of patients: patients with AP associated with normal spleen density and patients with AP associated with DROSD. Clinically, the APACHE II score is a widely used scoring system that can quantitatively evaluate the severity of AP, and serum calcium is a common laboratory test that can dynamically evaluate the severity of AP [37,40,41]. Our analysis showed that the APACHE II score was higher, and the concentration of serum calcium was lower in the reduction group compared with the control group (Table 3), suggesting that the condition of AP patients associated with DROSD was more serious. To verify further our clinical analysis results, we induced MAP and SAP in rats by infusing different concentrations of sodium taurocholate into the biliopancreatic duct, as described previously [28,29]. In this animal experiment, the severity of the models was evaluated based on pathological scoring of the pancreatic tissue, and the spleen density of the rats was measured [34,35]. Our experimental results showed that the pathological score was higher in the SAP group than in the sham and MAP groups (Figure 3b), whereas the spleen density was lower in the SAP group (Figure 3c). These results again demonstrated that DROSD was an independent indicator of AP severity. Clinically, accurate and prompt prediction of severe AP would be of great value in helping clinicians to classify patients for the appropriate level of care and to guide early management and thus improve the treatment effects in AP [42]. Thus, several serum laboratory parameters have been studied individually as predictors of SAP, including serum calcium, IL-1, 6 and 8, C-reactive protein (CRP), haematocrit, polymorphonuclear elastase and others [37,41,43–45]. However, the accuracy of prediction has been limited because these levels are often influenced by fluid resuscitation, although they are readily available and inexpensive, and they have standardized reference ranges [45]. Quantitative and accurate scoring systems, such as the APACHE-II score, Ranson score etc., have also been used to predict the severity of AP [37,40,41,46]. However, one major limitation of the available scoring systems is that they are complex and frequently cumbersome to perform in clinical practice [47]. In contrast, imaging evaluation methods, such as the Balthazar CT grading system and modified CT severity index (MCTSI), are more popular clinically because they are obvious, routine and less affected by fluid resuscitation [24]. In the present study, the 37 cases of AP with DROSD included 24 SAP cases and 13 MSAP cases. We found not only that the condition of AP patients associated with DROSD was more serious, but we also found that the features of DROSD were visual and obvious. Furthermore, the spleen density had a spleen CT value as a basis for reference. Therefore, we believe that the DROSD could be an independent indicator of more severe conditions of AP, and it has a higher specificity. However, its sensitivity is relatively low due to not all SAP patients being complicated by DROSD.

There has been no report about the mechanism of the DROSD in AP. But in the previous studies about the splenic injury in AP, Yasuda et al. [48] indicated that splenic atrophy and weight loss occurred in rat experimental SAP. Thus, in order to explore the relationship between the DROSD and the splenic mass and volume in AP, we measured the mass and volume of the spleen in rats. We found that there were no differences in the average spleen mass in the sham, MAP and SAP groups (Figure 4a). However, the average spleen volume in the SAP group was significantly larger than that in the sham and MAP groups (Figure 4b). These findings indicated that the reduction in spleen density in SAP rats was related to the increase in spleen volume. Although previous studies of spleen injury in AP were weak, the study of the mechanism of liver injury in AP was relatively adequate. For example, in animal studies, Zhang XP et al. [49] found that SAP could cause the liver mild swelling, dilated intrahepatic vessels dilated and congested hepatic sinusoids; Arita et al. [50] showed that the liver could present with increased vascular permeability, microvascular disturbances and abnormal haemoperfusion when SAP was complicated by liver damage, and these factors could cause reduced liver density on CT. It was therefore of interest to observe whether there was also abnormal spleen haemoperfusion in the present study. Our sonograms from colour Doppler ultrasound showed that the blood flow signals of the spleen did not differ between the sham group and MAP group of AP rats, and all of them presented with clustered and locally dense blood flow signals (Figure 5a); however, the SAP group showed a punctate or linear pattern of blood flow signals (Figure 5a), suggesting that spleen haemoperfusion in the SAP group was reduced. Furthermore, on ultrasound contrast imaging, no significant difference in visible signal intensity of the spleen was observed between the sham and MAP groups (Figure 5b); however, a lower visible signal intensity of the spleen was observed in the SAP group (Figure 5b). These findings further indicated that the spleen could show abnormal haemoperfusion in SAP. However, whether the abnormal haemoperfusion observed in our animal study would occur in AP patients was not clear because the course and distribution of the splenic vessels in rats are somewhat different from those in humans. We will study this issue in future studies under the informed consent of patients. Furthermore, Sirotakova et al. [51] showed that the splenic sympathetic nerve, accompanied by the splenic artery entering the spleen, regulates spleen haemoperfusion and the immune function of the spleen lymphocytes; MacNeil BJ et al. [52] found that the relationship between the electrophysiological activity of the spleen and endotoxins occurred in a dose-dependent manner. That is, with more endotoxins, we could see higher activity intensity of the spleen sympathetic nerve, a shorter incubation period and less haemoperfusion of the spleen. Herein, we hypothesized that, similar to injury of the liver in SAP, the spleen might also experience microvascular disturbances and abnormal haemoperfusion with permeation and attack by trypsin, inflammatory cytokines, endotoxins, and other compounds, which are produced in the process of SAP and then cause the spleen volume to increase and all of these factors might cause the spleen to show DROSD on CT imaging.

Additionally, some scholars have speculated that the mechanism of DROSD in AP might be due to high blood lipids, which can result in the deposition of lipids in the internal organs of these patients [10]. Unfortunately, relevant evidence-based medicine has been lacking. In the present study, we collected the TC and TG values of 377 patients with AP (including 37 cases of AP associated with DROSD) in our hospital, and we analysed the correlation between DROSD and blood lipids by multivariate logistic regression analysis for the first time. This analysis revealed that TC and TG did not differ between the control and reduction groups of patients with AP (Figure 7a and b), suggesting that DROSD was not attributable to high blood lipid levels in these patients. Furthermore, we used GSI to process the imaging analysis to determine whether DROSD was caused by the deposition of lipids or not. GSI is a type of imaging technology that can change the attenuation coefficient of X-rays in the material into the corresponding image and can provide not only monochromatic CT images but also spectral CT curves for the analysis of organ components [53–55]. For example, Hou and colleagues performed GSI to differentiate lung cancers from inflammatory masses, which have different histological structures, and they observed that the slopes of the spectral CT curves of these two masses differed [56]. Lv et al. [57] differentiated small hepatic haemangioma from small hepatocellular carcinoma using GSI. Du et al. [58] reported that the spectral CT curves of primary lesions of the gallbladder and metastatic retroperitoneal lymph nodes exhibited similar patterns because they originated from the same cells. Our analysis results showed that the spectral CT curves of the spleen and abdominal fat exhibited opposite trends in the patients with AP associated with DROSD (Figure 7c). Identical results were obtained for patients with AP associated with normal spleen density (Figure 7d). Thus, these findings further indicated that DROSD was not caused by the deposition of lipids in the spleen. Herein, a definite correlation between DROSD and blood lipids was not identified in the present study. Whether DROSD is related to blood lipids further requires a multicentre study.

Previous studies showed that splenocytes decreased in SAP, which resulted in peripheral lymphocytes reduction, leading to injury of immune function of the body [48]. Furthermore, the injury of the body's immune function was mainly due to the decreased activity of the helper T (TH) cell and suppressor T (TS) cell, but it had little relationship with B-lymphocytes [59]. Indeed, rat spleens were also examined by optical microscopy in the present study. Under optical microscopy, scattered fibroblast infiltration in the spleen was observed in the MAP group (Figure 7), whereas, in addition to fibroblast infiltration, we also observed a decrease in the number of spleen lymphocytes in the SAP group (Figure 7), suggesting that the cellular structure of the spleens of the rats was altered in AP, and lymphocytes were also involved. Subsequent TEM analysis revealed that the ultrastructure of spleen T-lymphocytes did not differ significantly in the MAP and SAP groups compared with the sham group (Figure 8a, b and d), whereas the ultrastructure of spleen B-lymphocytes were significantly different in these groups (Figure 8). In the MAP group, we observed apoptotic spleen B-lymphocytes (Figure 8b). However, in the SAP group, we observed necrotic and pyroptotic spleen B-lymphocytes (Figure 8c and d). These findings suggested that we could not ignore the impact of the changes in spleen B-lymphocytes when studying the changes of the immune function in SAP. All needs further study.

Because AP can lead to morphological and cellular changes in the spleen, we wanted to determine whether the immune function of the spleen would change or not. For the fact that the spleen is the maximal peripheral immune organ and crucial in regulating immune homoeostasis, it not only contains large numbers of B-lymphocytes, T-lymphocytes, macrophages and other immune cells, but it also synthesizes many types of hormones, such as serum conditioner, preparation solution and tuftsin [60]. Thus, to evaluate the immunological function of the spleen, we examined the percentages of IgM memory B-cells in the blood of volunteers and patients with AP. IgM memory B-cells, which require the spleen for their generation and/or survival, are a unique B-cell population that participates in the immune responses of the body by producing natural IgM antibodies [18,61]. Moreover, Di Sabatino A et al. [61] demonstrated that the recovery of splenic immune function in patients with Crohn's disease treated with infliximab was coincident with the restoration of IgM memory B-cells. Kruetzmann et al. [26] were unable to detect IgM memory B-cells in the blood when the spleen was absent [26]. Lammers et al. [62] indicated that the immunological function of the spleen could be analysed by evaluating IgM memory B-cells. In the present study, the percentage of IgM memory B-cells was obviously higher in the MAP group than in the healthy volunteer group (Figure 9c and d), suggesting up-regulation of the immunological function of the spleen. However, the percentage of IgM memory B-cells was significantly lower in the MSAP and SAP groups compared with the healthy volunteers (Figure 9c and d), suggesting down-regulation of the immunological function of the spleen. Thus, the immunological function of the spleen exhibited biphasic alteration in AP: up-regulation in mild AP and down-regulation in severe AP. In addition, we observed that the significantly lower percentage of IgM memory B-cells in AP patients with DROSD (similar to the value in the SAP group, as mentioned above) was lower than that in the AP patients with normal spleen density (Figure 9c and d), indicating down-regulation of the immunological function of the spleen in AP patients with DROSD. To verify further the changing trends in the immunological function of the spleen, we detected the tuftsin concentrations in the rat serum. Tuftsin, which is uniquely produced by the spleen, is a naturally occurring tetrapeptide that was originally described as an immune-regulating factor by Najjar in 1970, and it is a reliable marker of splenic immune function [38,39]. Zoli et al. [63] demonstrated that patients with a short bowel treated with long-term intravenous nutrition had impaired splenic immune function, accompanied by a decrease in tuftsin. Trevisani et al. [64] observed a decrease in tuftsin in cirrhosis when the immunological function of the spleen was impaired. In the present study, compared with the sham group, the concentration of serum tuftsin in the rat MAP group was not altered at 6 h after model induction, it was significantly increased at 12 and 24 h, and it returned to normal at 48 and 72 h (Figure 10c and d). However, in the SAP group, serum tuftsin was unchanged at 6 h and was significantly decreased at 12, 24, 48 and 72 h after model induction (Figure 10c and d). These results further demonstrated that the immune function of the spleen in AP exhibited a biphasic alteration, suggesting that the immune function of the spleen in AP also exhibited phasic alteration. Meanwhile, this is also consistent with the results of Shengji Yang et al. in whose study the splenic T-lymphocyte subsets (CD4, CD8 and CD4/CD8) were used to evaluted the changes of splenic immune function in AP [65]. However, limited by time, we did not study the reasons for the transient changes in tuftsin concentrations observed in rats. Nevertheless, numerous studies have suggested that TNF-α at a low concentration in serum could stimulate the secretion of tuftsin, but it could also inhibit the secretion of tuftsin at a high concentration [66–68]. Thus, we hypothesize that the reason for the transient changes in tuftsin concentrations were related to the concentration of TNF-α in serum during the course of AP. We will conduct further research on this aspect in our future experiments.

In conclusion, this study demonstrated for the first time that DROSD in AP was an independent indicator of AP severity. We observed that the presence of DROSD was related to increased spleen volume and decreased spleen haemoperfusion instead of blood lipids. Additionally, in AP, the damage to spleen B-lymphocytes was obvious, and the immunological function of the spleen exhibited a biphasic and phasic alteration, depending on AP severity. Furthermore, the immunological function of the spleen was down-regulated in AP patients with DROSD.

## Abbreviations

AP: acute pancreatitis
APACHE II: acute physiology and chronic health evaluation II
APC: allophycocyanin
CARS: compensatory anti-inflammatory response syndrome
CD: clusterdifferentiation
CRP: C-reactive protein
CTDI: computed tomography dose index
CT: computed tomography
DLP: dose–length product
DROSD: diffuse reduction of spleen density
GSI: gemstone spectral imaging
HE: haematoxylin and eosin
HLA-DR: human leucocyte antigen-DR
HU: hounsfield unit
IgM: immunoglobulin M
IgD: immunoglobulin D
IL: interleukin
LOS: length of stay
MAP: mild acute pancreatitis
MCTSI: modified CT severity index
MODS: multiple organ dysfunction syndrome
MSAP: moderately severe acute pancreatitis
OPSI: overwhelming post-splenectomy infection
PBMCs: peripheral blood mononuclear cells
PE: phycoerthrin
SAP: severe acute pancreatitis
SIRS: systemic inflammatory response syndrome
TC: total cholesterol
TG: triglyceride
TNF-α: tumour necrosis factor-α
TNFR-α: tumour necrosis factor receptor-α

## References

[1] Halonen K.I., Pettilä V., Leppäniemi A.K., Kemppainen E.A., Puolakkainen P.A., Haapiainen R.K. (2002) Multiple organ dysfunction associated with severe acute pancreatitis. Crit Care Med 30, 1274–1279 12072681 10.1097/00003246-200206000-00019

[2] Malmstrøm M.L., Hansen M.B., Andersen A.M., Ersbøll A.K., Nielsen O.H., Jørgensen L.N. (2012) Cytokines and organ failure in acute pancreatitis: inflammatory response in acute pancreatitis. Pancreas 41, 271–277 21956639 10.1097/MPA.0b013e3182240552

[3] Pandol S.J., Saluja A.K., Imrie C.W., Banks P. A. (2007) Acute pancreatitis: bench to the bedside. Gastroenterology 132, 1127–1151 17383433 10.1053/j.gastro.2007.01.055

[4] Hoque R., Farooq A., Ghani A., Gorelick F., Mehal, W.Z. (2014) Lactate reduces liver and pancreatic injury in Toll-like receptor- and inflammasome-mediated inflammation via GPR81-mediated suppression of innate immunity. Gastroenterology 146, 1763–1774 24657625 10.1053/j.gastro.2014.03.014 PMC4104305

[5] Boyer A., Chadda K., Salah A., Bonmarchand G. (2004) Thrombotic microangiopathy: an atypical cause of acute renal failure in patients with acute pancreatitis. Intensive Care Med 30, 1235–1239 15069598 10.1007/s00134-004-2272-y

[6] Sharif R., Dawra R., Wasiluk K., Phillips P., Dudeja, V., Kurt-Jones E. (2009) Impact of toll-like receptor 4 on the severity of acute pancreatitis and pancreatitis-associated lung injury in mice. Gut 58, 813–819 19201771 10.1136/gut.2008.170423

[7] Arenal Vera J.J., Said A., Guerro J.A., Otero M., Gil, I. (2008) Splenic infarction secondary to acute pancreatitis. Rev Esp Enferm Dig 100, 300–303 18662085 10.4321/s1130-01082008000500011

[8] Ray S., Mridha A.R. and Ahammed M. (2011) Diffuse splenic infarction in a case of severe acute pancreatitis. Am J Surg 201, e23–e25 21367360 10.1016/j.amjsurg.2010.03.020

[9] Hernani B.L., Silva P.C., Nishio R.T., Mateus H. C., Assef J. C., De Campos T., (2015) Acute pancreatitis complicated with splenic rupture: A case report. World J Gastrointest Surg 7, 219–222 26425272 10.4240/wjgs.v7.i9.219 PMC4582241

[10] Jiang X.Y., Bian J., Zhang C.Z., Wang S. S., Nie T. M., Zhang, L. (2014) Transient reduction of spleen density in acute pancreatitis: case reports and literature review. J Comput Assist Tomogr 38, 568–570 24651738 10.1097/RCT.0000000000000082

[11] Shen Y., Cui N., Miao B., Zhao E. (2011) Immune dysregulation in patients with severe acute pancreatitis. Inflammation 34, 36–42 20405190 10.1007/s10753-010-9205-4

[12] Ueda T., Takeyama Y., Yasuda T., Shinzeki M., Sawa H., Nakajima T. (2006) Immunosuppression in patients with severe acute pancreatitis. J Gastroenterol 41, 779–784 16988767 10.1007/s00535-006-1852-8

[13] Pereda J., Sabater L., Aparisi L., Escobar J., Sandoval J., Viña J. (2006) Interaction between cytokines and oxidative stress in acute pancreatitis. Curr Med Chem 13, 2775–2787 17073628 10.2174/092986706778522011

[14] Simovic M.O., Bonham M.J., Abu-Zidan F.M., Windsor J. A. (1999) Anti-inflammatory cytokine response and clinical outcome in acute pancreatitis. Crit Care Med 27, 2662–2665 10628606 10.1097/00003246-199912000-00009

[15] Fumeaux T. and Pugin J. (2002) Role of interleukin-10 in the intracellular sequestration of human leukocyte antigen-DR in monocytes during septic shock. Am J Respir Crit Care Med 166, 1475–1482 12406851 10.1164/rccm.200203-217OC

[16] Shen Y. and Cui N.Q. (2012) Clinical observation of immunity in patients with secondary infection from severe acute pancreatitis. Inflamm Res 61, 743–748 22466614 10.1007/s00011-012-0467-1

[17] Kylänpää ML1, Repo H. and Puolakkainen P.A. (2010) Inflammation and immunosuppression in severe acute pancreatitis. World J Gastroenterol 16, 2867–2872 20556831 10.3748/wjg.v16.i23.2867 PMC2887581

[18] Di Sabatino A., Carsetti R. and Corazza G.R. (2011) Post-splenectomy and hyposplenic states. Lancet 378, 86–97 21474172 10.1016/S0140-6736(10)61493-6

[19] Eraklis A.J., Kevy S.V., Diamond L.K., Gross R. E. (1967) Hazard of overwhelming infection after splenectomy in childhood. New Engl J Med 276, 1225–1229 6024340 10.1056/NEJM196706012762203

[20] Theilacker C., Ludewig K., Serr A., Schimpf J., Held J., Bögelein M. (2016) Overwhelming Postsplenectomy Infection: A Prospective Multicenter Cohort Study. Clin Infect Dis 62, 871–878. 26703862 10.1093/cid/civ1195

[21] Yasuda T., Takeyama Y., Ueda T., Takase K., Nishikawa J., Kuroda Y., (2002) Splenic atrophy in experimental severe acute pancreatitis. Pancreas 24, 365–372 11961489 10.1097/00006676-200205000-00007

[22] Hughes C.B., Henry J., Kotb M., Lobaschevsky A., Sabek O., Gaber A. O. (1995) Up-regulation of the TNF alpha mRNA in the rat spleen following induction of acute pancreatitis. J Surg Res 59, 687–693 8538166 10.1006/jsre.1995.1224

[23] Norman J.G., Fink G.W., Denham W., Yang J., Carter G., Sexton C. (1997) Tissue-specific cytokine production during experimental acute pancreatitis: a probable mechanism for distant organ dysfunction. Dig Dis Sci 42, 1783–1788 9286248 10.1023/a:1018886120711

[24] Banks P.A., Bollen T.L., Dervenis C., Gooszen H. G., Johnson C. D., Sarr M. G. (2013) Classification of acute pancreatitis—2012: revision of the Atlanta classification and definitions by international consensus. Gut 62, 102–111 23100216 10.1136/gutjnl-2012-302779

[25] Yang Z. and Li S. (2013) CT diagnosis of splenic diseases. Modern CT diagnostics (Tang G. Qin N., eds), pp. 927–950, China Medical Science Press, Beijing

[26] Kruetzmann S., Rosado M.M., Weber H., Germing U., Tournilhac O., Peter H. H. (2003) Human immunoglobulin M memory B cells controlling Streptococcus pneumoniae infections are generated in the spleen. J Exp Med 197, 939–945 12682112 10.1084/jem.20022020 PMC2193885

[27] Weller S., Braun M.C., Tan B.K., Rosenwald A., Cordier, C., Conley M. E. (2004) Human blood IgM "memory" B cells are circulating splenic marginal zone B cells harboring a prediversified immunoglobulin repertoire. Blood 104, 3647–3654 15191950 10.1182/blood-2004-01-0346 PMC2590648

[28] Cuthbertson C.M., Su K.H., Muralidharan V., Millar I., Malcontenti-Wilson C., Christophi C. (2008) Hyperbaric oxygen improves capillary morphology in severe acute pancreatitis. Pancreas 36, 70–75 18192884 10.1097/mpa.0b013e3181485863

[29] Bai X., Song Z., Zhou Y., Pan S., Wang F., Guo Z. (2014) The apoptosis of peripheral blood lymphocytes promoted by hyperbaric oxygen treatment contributes to attenuate the severity of early stage acute pancreatitis in rats. Apoptosis 19, 58–75 24101212 10.1007/s10495-013-0911-x

[30] Najjar V.A. and Constantopoulos A. (1972) A new phagocytosis-stimulating tetrapeptide hormone, tuftsin, and its role in disease. J Reticuloendothel Soc 12, 197–215 4116440

[31] Ying M., Leung G., Lau T.Y., Tipoe G. L., Lee E. S., Yuen Q. W. (2012) Evaluation of liver fibrosis by investigation of hepatic parenchymal perfusion using contrast-enhanced ultrasound: an animal study. J Clin Ultrasound 40, 462–470 22847895 10.1002/jcu.21969

[32] Feng J., Chen S.B., Wu S.J., Sun P., Xin T. Y., Chen, Y. Z. (2015) Quantitative analysis of contrast-enhanced ultrasonography in acute radiation-induced liver injury: An animal model. Exp Ther Med 10, 1807–1811 26640553 10.3892/etm.2015.2764 PMC4665675

[33] Amoscato A.A., Babcock G.F. and Nishioka K., (1981) Analysis of contaminants in commercial preparations of the tetrapeptide tuftsin by high-performance liquid chromatography. J Chromatogr 205, 178–184 10.1016/s0021-9673(00)81826-9 6894150

[34] Schmidt J., Rattner D.W., Lewandrowski K., Compton C. C., Mandavilli U., Knoefel W. T. (1992) A better model of acute pancreatitis for evaluating therapy. Ann Surg 215, 44–56 1731649 10.1097/00000658-199201000-00007 PMC1242369

[35] Bai X., Sun B., Pan S., Jiang H., Wang F., Krissansen G. W. (2009) Down-regulation of hypoxia-inducible factor-1 alpha by hyperbaric oxygen attenuates the severity of acute pancreatitis in rats. Pancreas 38, 515–522 19287337 10.1097/MPA.0b013e31819cac24

[36] Gul N., Ozkorkmaz E.G., Kelesoglu I., Ozluk A. (2013) An ultrastructural study, effects of Proteus vulgaris OX19 on the rabbit spleen cells. Micron 44, 133–136 22726264 10.1016/j.micron.2012.05.010

[37] Bradley E.L. III (1993) A clinically based classification system for acute pancreatitis. Summary of the International Symposium on Acute Pancreatitis, Atlanta, Ga, September 11 through 13, 1992. Arch Surg 128, 586–590 8489394 10.1001/archsurg.1993.01420170122019

[38] Najjar V.A. and Nishioka K. (1970) "Tuftsin": a natural phagocytosis stimulating peptide. Nature 228, 672–67 5. 4097539 10.1038/228672a0

[39] Wu M., Nissen J.C., Chen E.I., Tsirka S. E. (2012) Tuftsin promotes an anti-inflammatory switch and attenuates symptoms in experimental autoimmune encephalomyelitis. PLoS One 7, e34933 22529957 10.1371/journal.pone.0034933 PMC3328491

[40] Robert J.H., Frossard J.L., Mermillod B., Soravia C., Mensi N., Roth M. (2002) Early prediction of acute pancreatitis: prospective study comparing computed tomography scans, Ranson, Glascow, Acute Physiology and Chronic Health Evaluation II scores, and various serum markers. World J Surg 26, 612–619 12098056 10.1007/s00268-001-0278-y

[41] Wu B.U. (2011) Prognosis in acute pancreatitis. CMAJ 183, 673–677 21422134 10.1503/cmaj.101433 PMC3071387

[42] Fisher J.M. and Gardner T.B. (2012) The "golden hours" of management in acute pancreatitis. Am J Gastroenterol 107, 1146–1150 22858994 10.1038/ajg.2012.91

[43] Papachristou G.I., Clermont G., Sharma A., Yadav D., Whitcomb D. C. (2007) Risk and markers of severe acute pancreatitis. Gastroenterol Clin North Am 36, 277–296 17533079 10.1016/j.gtc.2007.03.003

[44] Uhl W., Buchler M., Malfertheiner P., Martini M., Beger H. G. (1991) PMN-elastase in comparison with CRP, antiproteases, and LDH as indicators of necrosis in human acute pancreatitis. Pancreas 6, 253–259 1713669 10.1097/00006676-199105000-00001

[45] Koutroumpakis E., Wu B.U., Bakker O.J., Dudekula A., Singh V. K., Besselink M. G. (2015) Admission Hematocrit and Rise in Blood Urea Nitrogen at 24 h Outperform other Laboratory Markers in Predicting Persistent Organ Failure and Pancreatic Necrosis in Acute Pancreatitis: A Post Hoc Analysis of Three Large Prospective Databases. Am J Gastroenterol 110, 1707–1716 26553208 10.1038/ajg.2015.370

[46] Mounzer R., Langmead C.J., Wu B.U., Evans A. C., Bishehsari F., Muddana V. (2012) Comparison of existing clinical scoring systems to predict persistent organ failure in patients with acute pancreatitis. Gastroenterology 142, 1476–1482 22425589 10.1053/j.gastro.2012.03.005

[47] Papachristou G.I., Muddana V., Yadav D., O’Connell M., Sanders M. K., Slivka A. (2010) Comparison of BISAP, Ranson's, APACHE-II, and CTSI scores in predicting organ failure, complications, and mortality in acute pancreatitis. Am J Gastroenterol 105, 435–441 19861954 10.1038/ajg.2009.622

[48] Yasuda T., Takeyama Y., Ueda T., Takase K., Nishikawa J., Kuroda Y. (2002) Splenic atrophy in experimental severe acute pancreatitis. Pancreas 24, 365–372 11961489 10.1097/00006676-200205000-00007

[49] Zhang X.P., Zhang J., Ren Z., Feng G. H., Zhu W., Cai Y. (2008) Study on protecting effects of baicalin and octreotide on hepatic injury in rats with severe acute pancreatitis. World J Gastroenterol 14, 6551–6559 19030211 10.3748/wjg.14.6551 PMC2773345

[50] Arita T., Matsunaga N., Takano K., Hara A., Fujita T., Honjo K. (1999) Hepatic perfusion abnormalities in acute pancreatitis: CT appearance and clinical importance. Abdom Imaging 24, 157–162 10024402 10.1007/s002619900466

[51] Sirot'áková M., Schmidtová K. and Kocisová M. (2004) Butyrylcholinesterase-positive innervation of the spleen in rats. Acta Medica (Hradec Kralove) 47, 201–204 15568740

[52] MacNeil B.J., Jansen A.H., Greenberg A.H., Nance D. M. (2000) Effect of acute adrenalectomy on sympathetic responses to peripheral lipopolysaccharide or central PGE(2). Am J Physiol Regul Integr Comp Physiol 278, R1321–R1328 10801303 10.1152/ajpregu.2000.278.5.R1321

[53] Zhang D., Li X. and Liu B. (2011) Objective characterization of GE discovery CT750 HD scanner: gemstone spectral imaging mode. Med Phys 38, 1178–1188 21520830 10.1118/1.3551999

[54] Li A., Liang H., Li W., Wang Z., Pang T., Li J. (2013) Spectral CT imaging of laryngeal and hypopharyngeal squamous cell carcinoma: evaluation of image quality and status of lymph nodes. PLoS One 8, e83492 24386214 10.1371/journal.pone.0083492 PMC3875441

[55] Boll D.T., Patil N.A., Paulson E.K., Merkle E. M., Nelson R. C., Schindera S. T. (2010) Focal cystic high-attenuation lesions: characterization in renal phantom by using photon-counting spectral CT–improved differentiation of lesion composition. Radiology 254, 270–276 20032158 10.1148/radiol.09090068

[56] Hou W.S., Wu H.W., Yin Y., Cheng J. J., Zhang Q., Xu J. R. (2015) Differentiation of lung cancers from inflammatory masses with dual-energy spectral CT imaging. Acad Radiol 22, 337–344 25491737 10.1016/j.acra.2014.10.004

[57] Lv P., Lin X.Z., Li J., Li W., Chen K. (2011) Differentiation of small hepatic hemangioma from small hepatocellular carcinoma: recently introduced spectral CT method. Radiology 259, 720–729 21357524 10.1148/radiol.11101425

[58] Du H., Zhang H., Xu Y., Wang L. (2014) Neuroendocrine tumor of the gallbladder with spectral CT. Quant Imaging Med Surg 4, 516–518 25525590 10.3978/j.issn.2223-4292.2014.08.04 PMC4256248

[59] Shugaev A I and Shabanova L F (1992) The effect of hemosorption on the immunological status of patients with acute destructive pancreatitis. Vestnik khirurgii imeni II Grekova 150, 78–80 8379091

[60] Mebius R.E. and Kraal G. (2005) Structure and function of the spleen. Nat Rev Immunol 5, 606–616 16056254 10.1038/nri1669

[61] Di Sabatino A., Rosado M.M., Cazzola P., Biancheri P., Tinozzi F. P., Laera M. R. (2008) Splenic function and IgM-memory B cells in Crohn's disease patients treated with infliximab. Inflamm Bowel Dis 14, 591–596 18240280 10.1002/ibd.20374

[62] Lammers A.J., de Porto A.P., Bennink R.J., van Leeuwen E. M., Biemond, B. J., Goslings J. C. (2012) Hyposplenism: comparison of different methods for determining splenic function. Am J Hematol 87, 484–489 22488175 10.1002/ajh.23154

[63] Zoli G., Corazza G.R., Wood S., Bartoli R., Gasbarrini G., Farthing M. J. G. (1998) Impaired splenic function and tuftsin deficiency in patients with intestinal failure on long term intravenous nutrition. Gut 43, 759–762 9824601 10.1136/gut.43.6.759 PMC1727358

[64] Trevisani F., Castelli E., Foschi F.G., Parazza M., Loggi E., Bertelli M. (2002) Impaired tuftsin activity in cirrhosis: relationship with splenic function and clinical outcome. Gut 50, 707–712 11950821 10.1136/gut.50.5.707 PMC1773217

[65] Yang S-J, Sun Y. and Cheng Y. (2004) Changes of splenic T-lymphocyte subsets in rats with acute pancreatitis. Chinese J Curr Adv Gen Surg 7, 356–357

[66] Zhu A., Jiang H., Liu L., Xu J., Qiao H. (2000) Effects of tumor necrosis factor-α on serum tuftsin level in rats with and without spleen. Chin J Hepatobiliary Surg 6, 365–367

[67] Eggermont A.M., Manusama E.R. and ten Hagen T.L. (1995) 1995–1996 Regional application of TNF alpha in the treatment of cancer: a preclinical-clinical interactive program. J Inflamm 47, 104–113 8913937

[68] Chu D.Z. and Nishioka K. (1990) Tuftsin increases survival in murine peritoneal carcinomatosis. J Biol Response Mod 9, 264–267 2160523

